# Exaptation of an ancient Alu short interspersed element provides a highly conserved vitamin D-mediated innate immune response in humans and primates

**DOI:** 10.1186/1471-2164-10-321

**Published:** 2009-07-16

**Authors:** Adrian F Gombart, Tsuyako Saito, H Phillip Koeffler

**Affiliations:** 1 Linus Pauling Institute, Department of Biochemistry and Biophysics, Oregon State University, Corvallis, Oregon, USA; 2 Department of Medicine, Division of Hematology/Oncology, Cedars-Sinai Medical Center, David Geffen School of Medicine at UCLA, Los Angeles, California, USA

## Abstract

**Background:**

About 45% of the human genome is comprised of mobile transposable elements or "junk DNA". The exaptation or co-option of these elements to provide important cellular functions is hypothesized to have played a powerful force in evolution; however, proven examples are rare. An ancient primate-specific Alu short interspersed element (SINE) put the human *CAMP *gene under the regulation of the vitamin D pathway by providing a perfect vitamin D receptor binding element (VDRE) in its promoter. Subsequent studies demonstrated that the vitamin D-cathelicidin pathway may be a key component of a novel innate immune response of human to infection. The lack of evolutionary conservation in non-primate mammals suggested that this is a primate-specific adaptation. Evidence for evolutionary conservation of this regulation in additional primate lineages would provide strong evidence that the TLR2/1-vitamin D-cathelicidin pathway evolved as a biologically important immune response mechanism protecting human and non-human primates against infection.

**Results:**

PCR-based amplification of the Alu SINE from human and non-human primate genomic DNA and subsequent sequence analysis, revealed perfect structural conservation of the VDRE in all primates examined. Reporter gene studies and induction of the endogenous *CAMP *gene in Rhesus macaque peripheral blood mononuclear cells demonstrated that the VDREs were conserved functionally. In addition, New World monkeys (NWMs) have maintained additional, functional steroid-hormone receptor binding sites in the AluSx SINE that confer retinoic acid responsiveness and provide potential thyroid hormone receptor binding sites. These sites were less well-conserved during human, ape and Old World monkey (OWM) evolution and the human *CAMP *gene does not respond to either retinoic acid or thyroid hormone.

**Conclusion:**

We demonstrated that the VDRE in the *CAMP *gene originated from the exaptation of an AluSx SINE in the lineage leading to humans, apes, OWMs and NWMs and remained under purifying selection for the last 55–60 million years. We present convincing evidence of an evolutionarily fixed, Alu-mediated divergence in steroid hormone nuclear receptor gene regulation between humans/primates and other mammals. Evolutionary selection to place the primate *CAMP *gene under regulation of the vitamin D pathway potentiates the innate immune response and may counter the anti-inflammatory properties of vitamin D.

## Background

The pioneering work of Britten and colleagues showed that eukaryotic genomes contain significant amounts of repetitive DNA [1,2]. They theorized that repetitive DNA might provide binding sites for transcriptional factors, thus influencing gene expression patterns [3]. Furthermore, it was postulated that the movement of repetitive sequences in the genome could generate a source of evolutionary variation for gene expression [4]. The human genome project has revealed that 44% of the human genome is comprised of mobile transposable elements [5]. Changes in regulation of gene expression by these elements may play an important role in the evolution of human and primate-specific responses to infectious disease.

The primate-specific Alu family of mobile, middle repetitive short-interspersed elements (SINEs) constitutes about 10% of the human genome [6,7]. They have an increased concentration of transcription factor binding sites and examples of their potential to control gene transcription both positively and negatively have been described [8-11]. We recently identified the insertion of an AluSx SINE in the promoter of the human cathelicidin antimicrobial peptide (*CAMP*) gene that provides an essential cis-element that may be crucial for an effective innate immune response in humans [12]. The element provides a perfect consensus sequence for binding by the vitamin D receptor (VDR) and confers vitamin D-responsiveness to the *CAMP *gene in a number of tissues and cell types [12-16]. Prior studies using both deletional and site-directed mutagenesis of the VDRE located in the AluSx of the human CAMP promoter demonstrated that it is sufficient and essential for induction of the CAMP gene by VDR [12-14,16]. Furthermore, chromatin immunoprecipitation showed binding of the VDR to this site [12].

*In vitro *studies demonstrated that activation of the vitamin D pathway by the *Mycobacterium tuberculosis *(Mtb) 19-kDa lipoprotein via TLR2/1 leads to induction of *CAMP *and a potential innate immune response against Mtb infection [17,18]. Furthermore, injury to the skin induces TGFβ-mediated activation of the vitamin D pathway, induction of *CAMP *and activation of TLR2 and CD14 expression [19]. This enables keratinocytes to recognize pathogens and protect the wound from infection [19]. Taken together, these studies argue that the vitamin D-cathelicidin pathway is a key component of a novel innate immune response to infection.

We demonstrated that regulation of the *CAMP *gene by VDR and its ligand 1,25(OH)_2 _vitamin D_3 _is not evolutionarily conserved in mice, rats or dogs because the promoters of their genes lack a VDRE [12]. The AluSx SINE containing the VDRE was present in the promoters of both chimps and humans suggesting that this immune response is a primate-specific adaptation [12]. Evidence for the evolutionary conservation of this regulation in additional primate lineages would provide strong evidence that the TLR2/1-vitamin D-cathelicidin pathway evolved as a biologically important immune response mechanism for protecting human and non-human primates against infection.

We hypothesized that the AluSx containing the VDRE would be evolutionarily conserved in humans, apes, Old World monkeys (OWMs) and New World monkeys (NWMs). To test this, we analyzed the sequence and function of the Alu SINEs amplified from genomic DNA of five apes, three OWMs and four NWMs. We determined that the VDRE is conserved in all three groups of primates and lacking in prosimians. Also, we discovered that NWMs may have maintained additional, functional steroid-hormone receptor binding sites in the AluSx SINE that could confer retinoic acid (RA) and thyroid hormone (TH) responsiveness; however, these sites were less highly conserved during human, ape and OWM evolution.

Comparisons of genomes ranging from ancient fish to modern humans have provided circumstantial evidence that the exaptation [20] or co-option of TEs to serve important cellular functions has been a powerful force in evolution [21,22]; however, proven examples are rare. Our study provides strong evidence that exaptation of an AluSx SINE provides a novel, biologically important innate immune response via the vitamin D-pathway that is evolutionarily conserved in humans and non-human primates, but absent in other mammals.

## Results

### Alu sequences are present in the *CAMP *promoters of Old and New World Monkeys

**Figure 1:**
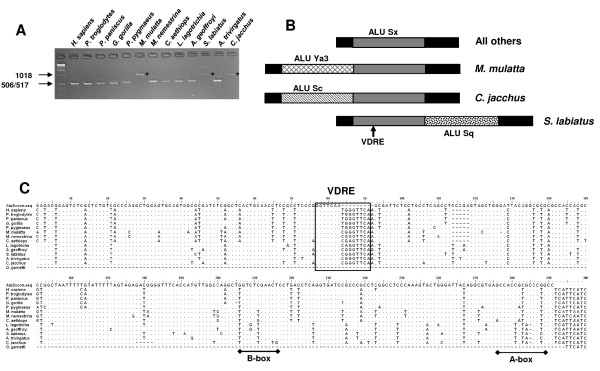
**Conservation of the VDRE-containing AluSx SINEs in humans, apes, OWMs and NWMs**. A) Amplification of the AluSx SINEs from a panel of non-human primates and humans. Products of the expected size were found in all but three primates. The increase in fragment sizes for *M. mulatta*, *S. labiatus *and *C. jacchus *was due to an additional Alu insertion. B) Schematic indicating the position and type of Alu insertion that was identified from sequencing the PCR products amplified in panel A. The location of the VDRE is indicated by the arrow. C) The nucleotide sequences of each AluSx SINE amplified in panel A were aligned with the AluSx consensus sequence [74]. The positions of the A- and B-boxes are indicated by an underline and the position of the VDRE is outlined by a box.

PCR products were amplified from each primate DNA except *Lemur catta *(Fig. 1A). The PCR products were approximately the same size for each sample except for *Macaca mulatta*, *Saguinus labiatus*, and *Callithrix jacchus*. For each of these primate samples the PCR product was approximately 300 bp larger. Sequencing of the shorter products showed that they were SINEs of the AluSx subfamily (Figures 1B and 1C). In the other three primate samples the AluSx SINE was present, but additional, independent insertional events occurred at the locus with SINEs present in the 5'- or 3'-termini of the shared AluSx SINE (Figure 1B). In *M. mulatta*, the Alu belonged to the Y subfamily and for the other two primates the Alu belonged to the S subfamily (Sc, *C. jacchus *and Sq, *S. labiatus*). The Alu element has a bipartite structure with the 5'-half containing the RNA polymerase-III promoter (A and B boxes) and the 3'-terminus possessing a run of A-nucleotides [7]. The AluSx is inserted 3'-to-5' with respect to the *CAMP *gene transcriptional start site (Figure 1B). The subsequent insertions of the AluSc and AluSq are 3'-to-5', as well (Figure 1B).

The VDRE is located in the 3'-half of the Alu-element. Alignment with the AluSx consensus sequence indicates the VDRE was formed by the duplication of the sequence 5'-CGGGTTCAA-3' (Fig. 1C). This resulted in the positioning of two direct repeats of 5'-GGTTCA-3' separated by a 3-nucleotide spacer and is an ideal VDRE [23].

A search of the trace archive data base at NCBI using the human *CAMP *coding region, identified overlapping sequences that included the 5'-promoter region of the *Otolemur garnetti *(a prosimian) *CAMP *gene (data not shown). Sequence alignments showed that the Alu-element and VDRE found in the other primates was absent in the *O. garnetti *(Figure 1C). This indicates that prosimians lack an Alu with a VDRE in their *CAMP *promoters.

### Functional conservation of VDRE activity

**Figure 2:**
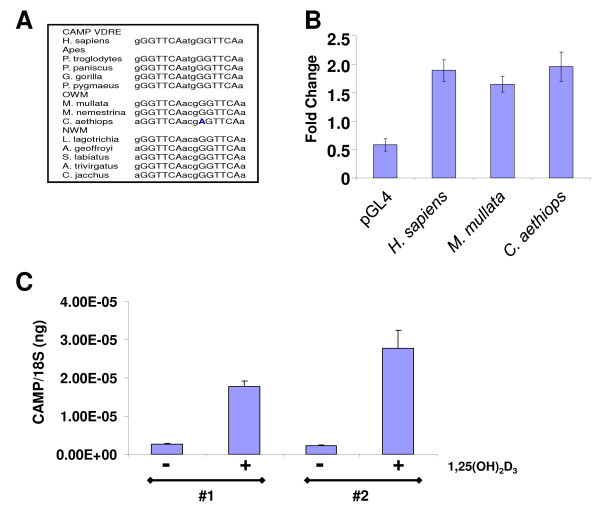
**Structural and functional conservation of the VDREs in primate promoters**. A) The nucleotide sequence of each primate VDRE is aligned to demonstrate the high degree of conservation of the direct repeats (upper case) and 3-bp spacer (lower case). *C. aethiops *contained a G-to-A change in the second direct repeat otherwise all the direct repeats were identical. B) To determine if primate VDREs were activated by 1,25(OH)_2_D_3_, the amplified Alus for *H. sapiens*, *M. mulatta *and *C. aethiops *were subcloned into the pGL4 luciferase reporter vector. The constructs were co-transfected into U937 cells with phTKRL (Promega) to control for efficiency. The change in expression is represented as fold-change comparing vehicle treated to 1,25(OH)_2_D_3 _treated cells. C) Mononuclear cells were isolated from the peripheral blood of two individual *M. mullata *and treated with either vehicle or 100 nM 1,25(OH)_2_D_3 _for 48 h. The expression level of CAMP mRNA was determined by QRT-PCR and normalized to 18S levels. The data are represented as ng of CAMP per ng of 18S.

The VDRE sequence was perfectly conserved in all primates with only *Cercopithecus aethiops *containing a G to A change in the first nucleotide position of the first direct repeat (Figures 1C and 2A). This change would not be expected to affect binding of the VDR to this site. Indeed, the Alu SINEs from *Homo sapiens*, *M. mulatta*, *C. aethiops *responded similarly to vitamin D treatment with increased luciferase activity as compared with the empty vector indicating that the base pair difference or insertion of an additional SINE did not affect binding of the VDR to the VDRE (Figure 2B).

### Induction of the *CAMP *gene by 1,25(OH)_2_D_3 _in *R. mulatta *mononuclear cells

To determine if the endogenous *CAMP *gene could be induced by 1,25(OH)_2_D_3 _in cells from a non-human primate, we isolated mononuclear cells (MNCs) from the peripheral blood of two individual *M. mulatta *and treated these cells with either vehicle (ethanol) or 100 nM 1,25(OH)_2_D_3 _for 48 h. Analysis by QRT-PCR showed that the *CAMP *gene was induced 6.8- and 12.2-fold. These results demonstrate that it is possible to induce the endogenous *CAMP *gene in non-human primates with 1,25(OH)_2_D_3 _(Figure 2C). Also, we detected the protein, CAP18, encoded by the CAMP gene in the plasma of the two *M. mulatta *(189 and 253 ng/ml) indicating that the protein is secreted into the blood as reported for humans [24].

### Other potential steroid hormone receptor response elements in the Alu-element are not functional

**Figure 3:**
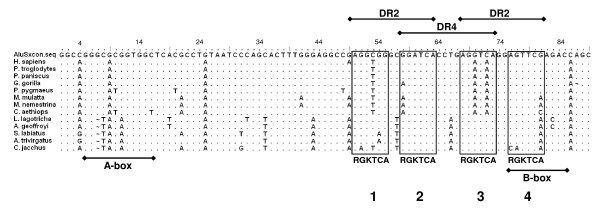
**Potential retinoic acid and thyroid hormone receptor binding sites conserved in AluSx of NWMs**. The nucleotide sequence (1–89) of the AluSx of each primate was aligned with the consensus AluSx sequence. The location of four direct repeats is indicated within each box with the consensus sequence indicated below each. The potential retinoic acid receptor binding sites (DR2) and the thyroid hormone binding site (DR4) are indicated above the boxes. The locations of the A- and B-boxes of the Alu are indicated below the sequences. The G-to-A and C-to-A change in positions three and five in the third direct repeat occurs in the human, all apes and OWMs, but not NWMs. These changes would likely abrogate binding of TR and RAR to the DR4 and the second DR2, respectively. All NWMs acquired a G-to-A change in the sixth position of the fourth direct repeat thus creating a potentially better DR2 for retinoic acid receptor binding. The third direct repeat in NWMs was unchanged from the consensus Alu sequence and would provide an ideal direct repeat providing a potentially functional DR4 and DR2.

The ability of Alu-elements to confer estrogen, retinoid and thyroid hormone responsiveness to genes has been described [25-27]. The response elements conferring binding of these receptors are located between the A- and B-boxes of the 5'-half of the Alu-element (Figure 3). In AluSx SINEs, four 6-bp direct repeats are arranged such that the first two repeats form a potential retinoic acid receptor (RAR) binding site (DR2) and the second and third repeats form a potential thyroid hormone receptor (TR) binding site (DR4). The third and fourth repeats form a second RAR binding site (Figure 3).

An AluSx SINE resides within the first 500 bp of the human *MPO *gene promoter [26]. The first DR2 was unable to bind RAR, but the second potential DR2 bound RAR and was responsive to retinoic acid [26]. Also, the DR4 was responsive to thyroid hormone [26]. Alignment of our primate sequences against the AluSx consensus sequence revealed that two nucleotide positions in the third direct repeat were changed from G-to-A and C-to-A for all hominids and Old World monkeys, but were unchanged in all the New World monkeys (Figure 3). These changes potentially could impair binding of either the RAR or TR and prevent activation of the *CAMP *gene by retinoic acid or thyroid hormone. These changes were not present in the third direct repeat of the *MPO *AluSx which is responsive to both steroid hormones [26].

**Figure 4:**
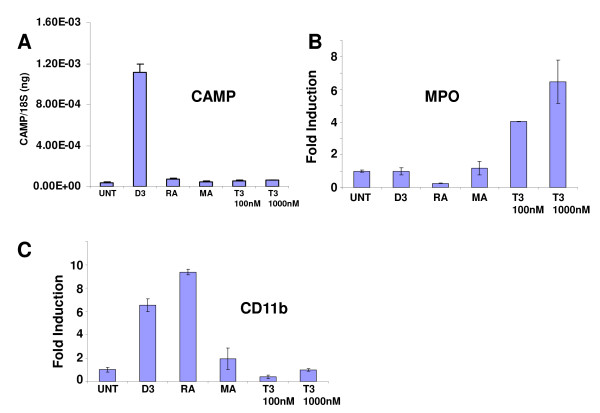
**The human CAMP gene does not respond to retinoic acid or thyroid hormone treatment**. The human myeloid cell line NB4 was treated with either vehicle (UNT), 1,25(OH)_2_D_3 _(D3), retinoic acid (RA), methoprene acid (MA) or thyroid hormone (T3). Expression of either A) CAMP, B) MPO or C) CDllb was determined by QRT-PCR and normalized to 18S rRNA.

To test the effect of these changes on *CAMP *gene expression, we treated the human myeloid cell line NB4 with vehicle, 1,25(OH)_2_D_3_, all trans retinoic acid (ATRA) and thyroid hormone (T3). As expected 1,25(OH)_2_D_3 _strongly induced *CAMP *expression, but neither ATRA nor T3 induced *CAMP *(Figure 4). To determine that the ligands and their respective receptors were functioning in these cells, we tested *ITGAM *(CDllb) and *MPO *expression as positive controls. As expected, *ITGAM *levels increased and *MPO *levels decreased with ATRA treatment [26,28,29] and *MPO *levels increased with T3 treatment [26] (Figure 4). These results suggest that the human, ape and OWM *CAMP *promoter Alu-elements have lost the ability to respond to these steroid hormones.

### NWM *CAMP *gene responds to retinoic acid

NWMs are resistant to vitamin D, sex steroids and glucocortocoids [30,31]. They are characterized by high circulating levels of these hormones. Interestingly, NWMs do not appear to have resistance to RA or T3 [32].

The third direct repeat in the NWM Alu-elements is identical to that in the consensus AluSx sequence (Figure 3). Furthermore, the fourth direct repeat acquired a G to A change in the sixth nucleotide of the direct repeat (Figure 3) and would be predicted to make the second DR2 a better binding site for RAR as it is identical to the site in the *MPO *gene that responds to RA [26]. Furthermore, the changes would make the DR4 a better binding site for the TR and is identical to the *MPO *DR4 that responds to TR [26].

**Figure 5:**
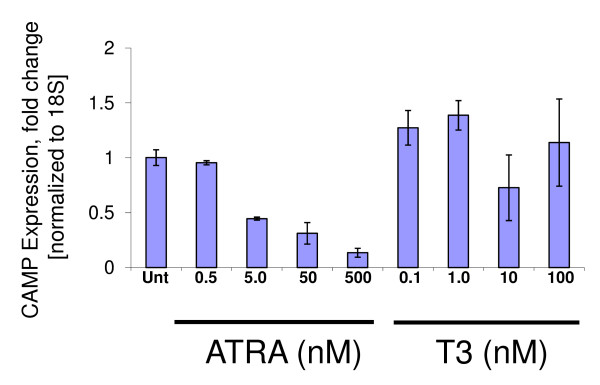
**The NWM CAMP gene responds to retinoic acid**. The marmoset (*C. jacchus*) B-cell line B95-8 was treated with increasing doses of either ATRA (0.5 to 500 nM) or T3 (0.1 to 100 nM) for 24 h. Relative fold-change compared with untreated cells was determined for CAMP gene expression (normalized to 18S rRNA) using QRT-PCR. CAMP gene expression decreased for ATRA treatment in a dose-dependent fashion. CAMP gene expression was unaffected by T3 treatment.

We hypothesized that these cis-elements in NWMs may confer responses to either ATRA or T3. To test this, we treated B95-8 cells, a lymphoblastoid B-cell line derived from the vitamin D-resistant NWM *C. jacchus*, with either 1,25(OH)_2_D_3_, ATRA or T3. As expected, the *CAMP *gene did not respond to vitamin D, but we observed a reproducible, dose-dependent reduction in *CAMP *gene expression with ATRA, but no changes in expression with T3 (Figure 5). These data indicate that the NWM gene has retained the ability to respond to ATRA while the OWM, apes and humans have not. The lack of response to T3 suggests that the NWM *CAMP *gene may not respond to this hormone or that other cell types or tissues need to be examined.

## Discussion

Early in primate evolution (about 60 million years ago), the major groups of Hominidae (humans and other apes), OWMs, NWMs and prosimians (lemurs and lorises) evolved independently and the origin and amplification of Alu elements was concomitant with this primate radiation [7]. Examples of Alu elements implicated in gene regulation have been identified [9-11,21,22,26,33-36]. Identification of convincing examples of evolutionarily-fixed, novel regulatory patterns requires evidence of: 1) a known transposable element (TE) sequence in the region of the gene; 2) the TE sequence affecting regulation of the nearby gene; 3) the gene having some function and 4) the TE having been present long enough to be fixed [10,34]. For some of the above examples, it was demonstrated that the Alu elements are conserved in humans, apes and/or OWMs, but was not examined in NWMs; therefore, the last requirement was only partially addressed [37].

Previously, we and others established the first three criteria for the *CAMP *gene [12,13]. Here we provide convincing evidence for the last requirement. We demonstrate that the TE (AluSx SINE) acquired a VDRE through a duplication event and has undergone approximately 60 million years of purifying selection during the primate radiation to become fixed in the genomes of present-day humans, apes, OWMs and NWMs (Figure 1). The distribution of this Alu in the different lineages is consistent with current primate phylogeny and the ancient age of this class of Alu [7]. Furthermore, we have shown that, as with humans, the non-human, primate VDREs are functional both *in vitro *and *in vivo *(Figure 2). Regulation of the *CAMP *gene by vitamin D as conferred by the AluSx in its promoter meets each of the above criteria. This study demonstrates that exaptation of vitamin D-mediated gene regulation by an AluSx SINE provided a novel, biologically-important innate immune response that is conserved in humans and non-human primates, but not other mammalian species. It is a convincing example of an evolutionarily-fixed, Alu-mediated divergence in steroid hormone nuclear receptor gene regulation between humans/primates and other mammals.

During the course of evolution, each group of primates has acquired differences in the ability to respond to steroid hormones [38]. Surprisingly, NWM have circulating 1,25(OH)_2_D_3 _levels that are up to two orders of magnitude higher than those observed in OWM, apes and humans [30,38]. The NWMs are naturally resistant to 1,25(OH)_2_D_3 _due to the over expression of VDRE-binding proteins (VDRE-BP) that requires NWMs to maintain high levels of 1,25(OH)_2_D_3 _to displace it from binding sites [39,40]. Also, NWM are resistant to estrogen, testosterone and glucocortocoids [31,38], but remain sensitive to retinoic acid and thyroid hormone [32]. Although these differences in vitamin D physiology exist, we have demonstrated that the VDRE is conserved in all three groups of primates. Subclasses of AluS sequences provide a significant source of potential hormone response elements for retinoic acid and thyroid hormone receptors [36,41]. Interestingly, in NWMs these elements are well conserved in the *CAMP *promoter Alu and have acquired additional changes that make them potentially better binding sites for RAR and TR (Figures 3 and 5). On the other hand, these sites were altered during hominid, ape and OWM evolution and did not respond to RA or TH (Figures 3 and 4). Retaining responses to both vitamin D and these other steroid hormones may be necessary for a proper innate immune response in vitamin D-resistant, but RA- and TH-sensitive NWMs. The importance of these binding sites to the expression of the *CAMP *gene remains to be fully elucidated.

The importance of vitamin D and the active metabolite 1,25(OH)_2_D_3 _in immune function became apparent with the discovery of VDR expression in activated inflammatory cells [42,43]. Also, it was demonstrated that 1,25(OH)_2_D_3 _was produced by activated macrophages [44,45] and 1,25(OH)_2_D_3 _inhibited T-cell activation and proliferation [46-49]. Subsequently, it was shown that vitamin D has an inhibitory action on the adaptive immune system with a shift from Th1 to Th2 and T regulatory cells and inhibition of Th17 development [50-54]. Suppression of the adaptive system and the anti-inflammatory effects of vitamin D are probably beneficial for conditions that involve autoimmunity [55]; however, it could prove detrimental for some infections [56,57].

The human *CAMP *gene is not induced consistently by pro-inflammatory stimuli [12,16,58-61]. Additionally, infection of macrophages with Mtb and other cell types with pathogens leads to the repression of the *CAMP *gene [18,62,63]. Acquisition of the VDRE by ancestral primates that likely possessed high levels of vitamin D like today's non-human primates [64] would have provided a pathway for induction of the *CAMP *gene in cells such as macrophages or epithelial barrier cells that are capable of activating the vitamin D pathway in response to infection or wounding [17,19]. The activation of the vitamin D pathway provides a way for human macrophages to prevent the suppression of the *CAMP *gene when activated with TLR2 or TLR4 ligands [65]. Thus, induction of the *CAMP *gene by 1,25(OH)_2_D_3 _provides a possible mechanism for primates to counteract pathogen-mediated suppression and modulate the immune response.

## Conclusion

We have demonstrated that the VDRE in the *CAMP *gene originated from the exaptation of an AluSx SINE in the lineage leading to humans, apes, OWMs and NWMs. It has remained under purifying selection for the last 55–60 million years. It is a convincing example of an evolutionarily fixed, Alu-mediated divergence in steroid hormone nuclear receptor gene regulation between humans/primates and other mammals.

The host immune response to serious infections is a delicate balancing act as bacterial clearance by an exuberant immune system often leads to self-induced immune damage, whereas a feeble immune response enables bacteria to persist and cause pathogen-induced diseases. We propose a possible model that explains how the vitamin D_3 _pathway may combat infection while minimizing damage to the host by its immune system. 1) TLR-activation by a pathogen activates production of 1,25(OH)_2_D_3 _and induction of VDR expression in monocytes [17]. 2) VDR-signaling increases production of hCAP18/LL-37 protein (encoded by the *CAMP *gene) to kill the pathogen [17,66]; 3) this subsequently downregulates TLR in the monocytes [67] and 4) nuclear translocation of NF-κB/RelA is blocked, thus muting the response to LPS and the production of inflammatory cytokines [67-71]. Finally, hCAP18/LL-37 secreted by monocytes [12], binds circulating LPS and dampens the signal to innate immune cells as well as directly acting on the TLR-to-NF-kappaB pathway in monocytes/macrophages [72]. Evolutionary selection to place the *CAMP *gene under regulation of the vitamin D pathway may enable suppression of inflammation while potentiating innate immunity, thus maximizing the overall immune response to a pathogen and minimizing damage to the host.

## Methods

### Human and non-human primate genomic DNA samples

The human genomic DNA was provided by Dr. Carl Miller (Cedars-Sinai Medical Center, Los Angeles, CA). The *M. mulatta*, *C. jacchus *and *A. trivirgatus *genomic DNAs were provided by Dr. John Adams (University of California Los Angeles, Los Angeles, CA). The genomic DNA for *C. aethiops *was isolated from the COS-1 cell line. The remaining non-human primate genomic DNAs were purchased (Corriel Institute for Medical Research, Camden, NJ).

### PCR amplification, sequencing and cloning

The Alu sequences in the primate *CAMP *promoters were amplified using the following primers: Forward, 5'-gggcaacttgtcccttgcaaga-3' and Reverse, 5'-gggtgctcaagagcgttaaatccc-3'. The primers were located outside the *Alu *SINE in regions that showed the highest homology among human, chimp, mouse, rat and canine sequences. The primer sequences were identical to the human and chimp promoters.

PCR was performed with 100 ng of genomic DNA in 50 μl reactions with HotMaster Taq polymerase (Eppendorf AG, Hamburg, Germany). The primers were used at a final concentration of 300 nM. The annealing temperature was 50°C and 35 cycles of PCR were performed. The PCR products were purified from the reaction using a DNA Clean and Concentrator-5 spin column (Zymo Research, Orange, CA). The PCR products were sequenced using the PCR primers described above and the Big DyeTM Terminator v.3 cycle sequencing as instructed by the manufacturer (Applied Biosystems, Inc., Foster City, CA). The sequences were analyzed and aligned using the Bioedit Sequence Alignment Editor software (Tom Hall, Ibis Biosciences, Carlsbad, CA). The PCR products for the human, *M. mulatta *and *C. aethiops *Alu-elements were cloned into the pCR-2.1 Topo-TA vector (Invitrogen, Carlsbad, CA). These fragments were subcloned into pGL4.20 [Luc2/Puro] (Promega Corporation, Madison, WI).

### Cell Culture

The U937, HL-60, NB4 and B95-8 cells were grown in RPMI 1640 supplemented with 10% FBS and antibiotics. Cells were treated with either 1,25(OH)_2_D_3 _(1 or 100 nM), 500 nM all trans retinoic acid (ATRA) or thyroid hormone (T3, 10 or 100 nM) for 24 h. Whole blood from *M. mulatta *(two different animals) was obtained from the California National Primate Research Center (University of California, Davis, CA). The mononuclear cells (MNC) were isolated on a Ficoll gradient after lysis of the red blood cells. The MNC were cultured in RPMI 1640 with 10% FBS and antibiotics in the presence of either vehicle (95% ethanol) or 1,25(OH)_2_D_3 _(100 nM) for 48 h.

### Reporter Assays, RNA isolation and QRT-PCR

U937 cells were co-transfected with empty vector (pGL4.20) or vector containing the *Alu *SINE (1 μg) and phTKRL (0.1 μg) using Effectene reagent as described by the manufacturer (Qiagen, Chatsworth, CA). Cells were lysed and dual-luciferase assays performed as instructed by the manufacturer (Promgea Corporation).

Total RNA was isolated using Trizol Reagent according to the manufacturer (Invitrogen). The synthesis of the cDNA and QRT-PCR for the human *CAMP *gene and 18S rRNA were performed as described previously [12]. The expression of *ITGAM *(CDllb) and *MPO *were analyzed by QRT-PCR using SYBR-green as described previously [12,73].

## Authors' contributions

AFG: conceived the study, amplified, subcloned and sequenced the Alu SINEs from the primate genomic DNA, performed DNA alignments, performed the reporter assays, analyzed the data and drafted the manuscript. TS: treated tissue culture cells with the various steroid hormones, performed the QRT-PCR for CAMP, MPO and CDllb gene expression in the cell lines and primary monocytes from Rhesus macaque, and analyzed the data. HPK: participated in study design, coordination and analysis of data and helped draft the manuscript. All authors have read and approved the final manuscript.

## Authors' information

AFG is a Principal Investigator in the Linus Pauling Institute and Associate Professor in the Department of Biochemistry and Biophysics at Oregon State University, 2011 ALS Bldg, Corvallis, OR 97331-7305. E-mail: adrian.gombart@oregonstate.edu

TS is a postdoctoral researcher in the Linus Pauling Institute and Department of Biochemistry and Biophysics at Oregon State University, 2011 ALS Bldg, Corvallis, OR 97331-7305. E-mail: saito.tsuyako@oregonstate.edu

HPK is a Professor of Medicine at the David Geffen School of Medicine at UCLA, Director, Division of Hematology/Oncology at Cedars-Sinai Medical Center, 8700 Beverly Blvd, Los Angeles, CA 90048. E-mail: koeffler@cshs.org
